# Kratom withdrawal: Discussions and conclusions of a scientific expert forum^☆^

**DOI:** 10.1016/j.dadr.2023.100142

**Published:** 2023-03-15

**Authors:** Jack E. Henningfield, Marek C. Chawarski, Albert Garcia-Romeu, Oliver Grundmann, Norsyifa Harun, Zurina Hassan, Christopher R. McCurdy, Lance R. McMahon, Abhisheak Sharma, Mohammed Shoaib, Darshan Singh, Kirsten E. Smith, Marc T. Swogger, Balasingam Vicknasingam, Zachary Walsh, Daniel W. Wang, Marilyn A. Huestis

**Affiliations:** a PinneyAssociates, Bethesda, MD 20814, United States; b Department of Psychiatry and Behavioral Sciences, Johns Hopkins University School of Medicine, Baltimore, MD 21205, United States; c Departments of Psychiatry and Emergency Medicine, Yale School of Medicine, New Haven, CT 06519, United States; d Department of Medicinal Chemistry, College of Pharmacy, University of Florida, Gainesville, FL 32610, USA; e Centre for Drug Research, Universiti Sains Malaysia, Penang 11800, Malaysia; f Department of Pharmaceutics, College of Pharmacy, University of Florida, Gainesville, FL 32610, United States; g Translational Drug Development Core, Clinical and Translational Sciences Institute, University of Florida, Gainesville, FL 32610, United States; h Department of Pharmaceutical Sciences, Texas Tech University Health Sciences Center, Lubbock, TX 79430, United States; i Institute of Neuroscience, Medical School, Newcastle University, Newcastle Upon Tyne NE2 4HH, United Kingdom; j Real-World Assessment, Prediction, and Treatment Unit, National Institute on Drug Abuse Intramural Research Program, Baltimore, MD, United States; k Department of Psychiatry, University of Rochester Medical Center, Rochester, NY 14642, United States; l Department of Psychology, University of British Columbia, Kelowna, BC V1V 1V7, Canada; m Thomas Jefferson University, Philadelphia, PA 19144, United States

**Keywords:** Kratom, Mitragynine, Dependence, Withdrawal, Abuse potential, Animal, Human, Therapeutic treatment

## 1 Introduction and summary

Clarity on kratom's dependence and withdrawal potential increased substantially over the past decade with increased research funding from the US National Institute on Drug Abuse (NIDA, 2022), as well as the Centre for Drug Research, Universiti Sains Malaysia, and other organizations. Clarity is derived from new studies of kratom and its primary active alkaloid, mitragynine, from studies of physical dependence and withdrawal in animals and humans, United States (US) internet surveys, and Southeast Asia (SEA) field surveys. These investigations were the main focus of a global forum on kratom physical dependence and withdrawal potential (hereafter, The Forum) that brought together the investigators who conducted most of the new research to discuss their findings and provide recommendations that are the basis of this commentary.

Drs. Henningfield and Huestis identified and invited researchers who actively contributed to peer-reviewed studies evaluating kratom and mitragynine withdrawal to participate in the Forum and coauthor this commentary. Following self-introductions and opening thoughts on the topic, Dr. Henningfield offered a summary of key definitions and withdrawal assessment methods. Dr. Katz provided additional background on the history of laboratory methods for assessing withdrawal and physical dependence and an overview of the Yue et al. (2022) study that illustrated several of these concepts. To accommodate diverse schedules and time zones, the Forum was similarly conducted on two separate days with several participants attending both. This was followed by a general discussion that led to a variety of conclusions, and recommendations for further research as summarized below.

With respect to clinical and diagnostic terminology, the article is generally consistent with the approaches of the American Psychiatric Association (APA) and the World Health Organization's (WHO) diagnostic manuals, which use the term “dependence” to refer to the behavioral disorder often referred to as “addiction” or “compulsive use” but more recently employ the term “use disorder” in place of dependence (American Psychiatric Association, 2013; World Health Organization, 2019). APA and WHO refer to the term “withdrawal” as the disorder diagnosed on the basis of the symptoms that may emerge following discontinuation of chronic use.

Physical dependence and withdrawal were defined in The Forum consistent with the FDA's abuse potential guidance (Food and Drug Administration, 2017, p. 4) as follows: “Dependence refers to physical or psychological dependence. Physical dependence is a state that develops as a result of physiological adaptation in response to repeated drug use, manifested by withdrawal signs and symptoms after abrupt discontinuation or significant dose reduction of a drug.”… “The presence of physical dependence or tolerance does not determine whether a drug has abuse potential… However, if a drug has rewarding properties, the ability of that drug to induce physical dependence or tolerance may influence its overall abuse potential.”

Thus, as discussed further by FDA, “Many medications that are not associated with abuse, such as antidepressants, beta blockers, and centrally acting antihypertensive drugs, can produce physical dependence and/or tolerance after chronic use. However, if a drug has rewarding properties, the ability of that drug to induce physical dependence or tolerance may influence its overall abuse potential.” (FDA, 2017, p.4)

Thus, consistent with FDA's 2017 abuse potential guidance, physical dependence and withdrawal are neither necessary nor sufficient for the determination that a “use disorder” or behavioral “dependence” is present (See also O'Brien 2015). FDA's conceptualization is similar to leading pharmacology reviews (e.g., O'Brien 2015), in which physical dependence is evidenced by the observation of withdrawal symptoms emerging upon discontinuation of drug administration or following administration of an antagonist to the chronically administered substance (e.g., naloxone in the case of chronically administered opioids) (e.g., O'Brien, 2015).

Findings from recent studies of kratom/mitragynine withdrawal have been reviewed elsewhere (Henningfield et al., 2022) and in a more recently published animal study by Yue et al. (2022). Taken together, this research established that some kratom users reported withdrawal symptoms if they discontinue kratom use for a day or more and some reported feeling “dependent” or “addicted” (Prozialeck et al., 2019; Singh et al., 2014, 2016; Smith et al., 2022a, 2022b, 2021). Online surveys, qualitative data analyses, and case reports suggest that tolerance and withdrawal, or perceived addiction to kratom, is reported among US kratom users and that its severity is generally mild. The prevalence of withdrawal among kratom users in the US is not well established but was less than 10% overall in one survey, and higher among those who had opioid use disorder (OUD) and withdrawal that they managed with kratom (Garcia et al. 2020). For those who do report dependence or withdrawal, most find it mild, tolerable and self-manageable, with many using kratom to manage opioid withdrawal and symptoms of opioid use disorder (Boyer et al., 2008; Coe et al., 2019; Garcia-Romeu et al., 2020; Grundmann, 2017; Smith et al., 2021).

The fact that the online surveys to date are convenience samples of self-selected respondents limits their generalizability to the larger population of kratom consumers. However, the findings are consistent with observations in residential drug treatment programs (e.g., Smith and Lawson 2017) and field survey studies in SEA (Leong Bin Abdullah et al., 2021; Singh et al., 2014, 2020, 2018), and in recent global reviews of kratom use and effects (Henningfield et al., 2022; Karunakaran et al., 2022; Swogger and Walsh, 2018; WHO ECDD, 2021). Although kratom intake to self-manage OUD and opioid withdrawal is of particular interest in light of the opioid epidemic in the US, the aforementioned surveys in the US and Southeast Asia (SEA) suggest that the reason most consume kratom is for its diverse effects including as an alternative to caffeinated beverages for stimulating and alerting effects, and to improve overall health, functioning, and well-being. Recent research, suggests that such outcomes are consistent with the diverse effects are mediated by GABAergic, alpha-adrenergic, serotonergic, dopaminergic, and opioid receptors (Karunakaran et al., 2022; Obeng et al., 2021; Reeve et al., 2020; Sharma and McCurdy, 2021).

Animal studies of physical dependence and withdrawal, generally involve daily morphine administration following well established protocols that produce these effects (Harun et al., 2021, 2020; Hassan et al., 2021, 2020; Johari et al., 2021; Macko et al., 1972; Wilson et al., 2020), with one study (Yue et al., 2022) employing heroin instead of morphine. These animal studies then compared withdrawal following daily administration of mitragynine, and/or evaluated the effectiveness of mitragynine and lyophilized kratom tea in reducing withdrawal symptoms. The most typical paradigm for assessing efficacy in reducing withdrawal in these studies was to replace morphine administration with mitragynine or lyophilized kratom tea. In the study using heroin to establish physical dependence (Yue et al. 2022), precipitated withdrawal by the opioid antagonist naloxone was followed by comparison of mitragynine and heroin suppression of withdrawal – a model referred to as “cross-dependence” (Balster and Walsh, 2010; Himmelsbach and Andrews, 1943; Yue et al., 2022).

Taken together, these results suggest that withdrawal only occurs following high mitragynine intake, withdrawal signs are dissimilar and weaker than those following opioid withdrawal, and mitragynine and lyophilized kratom “tea” reduced morphine and/or heroin-related withdrawal signs (Harun et al., 2021, 2020; Hassan et al., 2021, 2020; Johari et al., 2021; Macko et al., 1972; Wilson et al., 2020; Yue et al., 2022). The differences in mitragynine and morphine withdrawal are consistent with their differing pharmacology given that mitragynine has mixed effects and mechanisms of action including partial opioid agonism, and alpha adrenergic, serotonergic and other receptor mediated effects (Behnood-Rod et al., 2020; Gutridge et al., 2020; Hassan et al., 2019; Hiranita et al., 2019; Hughes et al., 2022; Kruegel et al., 2019; Obeng et al., 2020; Patel et al., 2021; Qu et al., 2022; Suhaimi et al., 2021; Todd et al., 2020).

These physical dependence and withdrawal findings are also consistent with those following animal intravenous drug self-administration demonstrating that mitragynine reduces morphine and heroin self-administration (Hemby et al.. 2019; Yue et al., 2018) and human kratom use to manage opioid craving, withdrawal and OUD (e.g., Boyer et al. 2008, National Institute on Drug Abuse 2022, WHO ECDD 2021). Although the focus of most animal withdrawal studies was on mitragynine, other minor alkaloids and metabolites may contribute to the overall effects including physical dependence and withdrawal (Sharma and McCurdy, 2021). Marketed kratom products in which levels of alkaloids are increased beyond what is typically found in natural products may also differ in their potential for physical dependence, withdrawal and other effects.

Two clinical studies that systematically assessed withdrawal with the Subjective Opiate Withdrawal Scale (SOWS) and/or Clinical Opiate Withdrawal Scale (COWS) did not find evidence of withdrawal. In one, a Health Canada-approved study,198 participants (18-21 per study condition cohort) were assessed by the SOWS and COWS instruments and by adverse event monitoring for potential withdrawal signs and symptoms following daily intake of single doses of three kratom formulations at up to 29.6 mg mitragynine and following 15 consecutive daily doses (Huestis et al., 2022). There were no significant differences between placebo or any of the kratom dosing conditions in SOWS and COWS scores or adverse events related to withdrawal or abuse potential.

The other study included an assessment of a standardized dose of a kratom liquid on pain tolerance in the cold pressor test in “chronic” male participants with a mean of 6.1 years kratom use and self-administered kratom “multiple times per day” for 7 days before testing (Vicknasingam et al., 2020). After testing, withdrawal was assessed using the COWS and self-reports of potential discomfort. Pain tolerance was significantly increased for two hours following kratom administration as compared to placebo. No signs or symptoms of withdrawal were evident by COWS or self-reports.

More than 100 new study reports published in the past five years provide preliminary data informing our understanding of kratom's physical dependence and withdrawal potential, and kratom intake to manage withdrawal and use disorders for opioids, alcohol and stimulants (Henningfield et al. 2022; Prozialeck et al., 2019, 2021; Smith et al., 2022a, 2022b; Smith et al., 2021; Swogger et al., 2022).

Although we focus on mitragynine as the predominant alkaloid in kratom, the plant contains more than 40 alkaloids that may also contribute to kratom's effects and whose pharmacology we are just discovering. The ongoing opioid overdose emergency in the US and elsewhere gives urgency to applying what we know and researching what we still need to know because it is recognized that kratom is used by some to manage opioid withdrawal and as a path away from opioids (Giroir, 2018; National Institute on Drug Abuse, 2022; Prozialeck et al., 2019, 2021; WHO ECDD, 2021). However, kratom is not FDA-approved for this or any indication currently. This commentary does not address the relevance of these withdrawal related findings to overall abuse potential and safety which was discussed elsewhere (e.g., Henningfield et al. 2022; Prozialeck et al. 2019, 2021; WHO, ECDD, 2021).

## 2 Conclusions and recommendations

The Forum findings and conclusions were initially summarized by moderators Henningfield and Huestis, with subsequent input by Forum participants in the development of this commentary. The references to this commentary include the original research and recent review articles providing additional perspectives.

### 2.1 Conclusions related to kratom's alkaloids and pharmacology

Many kratom effects, including those related to dependence and withdrawal, are due in part to mitragynine; however, some of kratom's more than 40 other alkaloids and metabolites, such as 7-hydroxymitragynine, may also contribute to dependence and withdrawal and/or other effects. Mitragynine benefits in relieving morphine withdrawal in animal studies may reflect its non-opioid alpha-adrenergic, serotonergic, and other pharmacological effects, much as the alpha-adrenergic modulators lofexidine, which was FDA-approved for this indication in 2018, and clonidine, which is not FDA-approved for opioid withdrawal relief but was used for this purpose since the 1980s.

### 2.2 Conclusions related to withdrawal and physical dependence based on survey data

Self-report data derived from surveys, case reports and clinical encounters suggest convergence of the reasons for kratom use in the US, SEA, and other regions of the world. These include increasing general health and well-being, energy enhancement, analgesia, ameliorating symptoms of stress, anxiety and depression, enhancing physical and cognitive performance, and management of opioid dependence and withdrawal, as well as other addictions, particularly alcohol and psychostimulants.

One limitation of survey data is that there are no universally accepted criteria for distinguishing moderate from heavy kratom use based on frequency of use or actual grams of leaf material or mg mitragynine per dose. However, Forum participants agreed that the following observations are generally related to self-reported effects. Moderate daily kratom use (e.g., 2 g leaf material per dose, 1-2 times/day) of unadulterated and unaltered kratom leaf material or powder generally does not lead to a significant discontinuation-associated withdrawal syndrome. Withdrawal was reported, by some taking high amounts of kratom products (e.g., greater than 3 g of leaf material more than twice daily) for an extended period (i.e., likely exceeding 300 mg mitragynine per day).

Although withdrawal symptoms are more prevalent in people with higher and more frequent consumption, variability in self-reported withdrawal is substantial. For example, in observational research, withdrawal is not reliably evident even among heavy kratom consumers who consume three or more times per day. The reasons for this difference from morphine-like opioids, which have more reliable physical dependence producing effects may be based on pharmacological differences between opioids and kratom alkaloids. It is also possible that the variability is related in part to self-report accuracy limitations and lack of general consumer knowledge about mitragynine levels in their products. Reports of kratom withdrawal are also more likely in people with prior opioid use histories (Garcia-Romeu et al., 2020) suggesting that greater efforts need to be made to document opioid use histories in studies and surveys of kratom users (Smith et al., 2022a).

Regarding symptomatology, some of the reported kratom withdrawal symptoms are qualitatively similar to those of opioids (e.g., runny nose, muscular pain and diarrhea), whereas others are shared with sedatives and/or stimulants (e.g., lethargy, depressed mood and anxiety). Overall, kratom withdrawal symptoms are generally milder than observed with chronic frequent opioid, sedative, or stimulant users and generally more tolerable and self-manageable. Craving levels appear widely variable. An implication of the foregoing is that the use of opioids such as methadone and buprenorphine should not be the first line treatments for people claiming kratom withdrawal symptoms. Methadone and buprenorphine should only be used judiciously on an individual basis, especially if the person did not have a prior opioid use history because (1) these opioids might not be the optimal treatments and (2) such use may produce opioid tolerance and physical dependence, and possibly an opioid use disorder that was not already present (see discussion by Smith et al. 2022a).

### 2.3 Conclusions related to kratom intake to self-manage withdrawal and substance use disorders

US surveys and field studies in SEA indicate that a substantial portion of kratom consumers used kratom to self-manage opioid withdrawal and to achieve and sustain abstinence from opioids; however, the fraction of kratom consumers who use kratom for this reason varies widely across surveys. Consumption of kratom to manage withdrawal and/or “addictions” to alcohol, stimulants (e.g., methamphetamine, cocaine), and other drugs also were reported in most of the surveys cited in this commentary but are less well studied.

Although it is commonly assumed that kratom's effectiveness in relieving opioid withdrawal is evidence for its opioid effects, it is also consistent with its alpha-adrenergic effects that are similar to those of the non-opioids clonidine and lofexidine which can also relieve opioid withdrawal. Several kratom alkaloids also were found to have relatively weak G protein activation and beta arrestin recruitment and signaling effects as compared to opioids on pathways related to respiratory and other effects that may account in part for the differences with classic opioids (Henningfield et al., 2022; Qu, 2022).

### 2.4 Conclusions related to kratom physical dependence and withdrawal based on controlled clinical studies

The two clinical studies described above by Vicknasingam et al. (2020) that involved long term chronic multiple times per day kratom consumers, and Huestis et al. (2022) that employed kratom naïve subjects at lower doses did not observe signs of kratom withdrawal using the SOWS and COWS, adverse events, or spontaneous reports. These findings are consistent with the fact that kratom withdrawal is not reliably produced by moderate kratom consumption and not reliable with heavier kratom consumption as several of the participants in the Vicknasingam et al. study were heavier long term kratom consumers. Further such study is clearly needed to better understand the conditions under which mitragynine alone and/or in combination with other kratom alkaloids may produce physical dependence and withdrawal.

### 2.5 Animal studies related to kratom physical dependence and withdrawal, and use of kratom or mitragynine to reduce or prevent withdrawal signs

Across various animal models, results are generally consistent indicating mitragynine can produce physical dependence and withdrawal at high doses; however, the severity and possibly qualitative nature of the withdrawal is not the same as that produced by opioids, stimulants or sedatives. The animal studies of withdrawal are generally consistent with human survey and field studies finding substantially milder withdrawal symptoms overall following discontinuation of daily mitragynine as compared to discontinuation of daily morphine administrations. Animal studies also suggest that mitragynine is similar to buprenorphine and methadone in its apparent efficacy in alleviating morphine withdrawal signs.

## 3 Kratom research recommendations

It is important to better characterize the dose-response relationship and administration frequency between kratom and mitragynine intake and development of physical dependence and withdrawal severity. With regard to the characterization of kratom withdrawal, it may be helpful to employ assessment instruments, beyond the opioid validated SOWS and COWS and include measures for alcohol, caffeine, nicotine, and/or psychostimulant withdrawal. An important clinical goal of such research is to better characterize the symptoms that might serve to define kratom withdrawal and kratom use disorder for clinical diagnosis, and the eventual potential inclusion by APA and WHO in their diagnostic systems.

Recognizing that kratom's effects, including physical dependence, may be influenced by other kratom alkaloids and metabolites, suggests continuing research on these other substances is important. This includes research to better understand the potential dose-response relationships between kratom, and kratom alkaloids including but not limited to mitragynine, and their metabolites for treating opioid and other drug use disorders (e.g., stimulant and alcohol), as well as their potential abuse potential related effects (Table 1).

**Table 1:** Animal models for assessing physical dependence and withdrawal and potential treatment.^a^^,^^b^^,^^c^

Baseline physical dependence	Animals are made physically dependent by daily or multiple times per day drug administration or by including drug in food for at least 3 but up to 14 or more days.
Spontaneous withdrawal	Withdrawal signs are accessed following discontinuation of administration of the positive control (e.g., morphine) and test substance (e.g., mitragynine)
Precipitated withdrawal	Withdrawal signs are assessed following administration of a known antagonist (e.g., naloxone in the case of morphine receptor agonist opioids)
Single dose Suppression or Cross dependence	Evaluates a drug's potential to suppress precipitated withdrawal signs^d^

a Putative treatment drugs are assessed by these protocols to determine if they prevent withdrawal by administration following discontinuation of daily drug administration and if they can relieve withdrawal by administration following the emergence of withdrawal signs (Yue et al., 2022).

b Human studies with opioids rely upon volunteers with existing physical dependence who are typically stabilized on three times per day morphine. Their withdrawal discomfort is minimized by assessing early withdrawal signs following omission of one morphine dose and by using low doses of naloxone to precipitate withdrawal. The low dose naloxone “challenge” protocol also screens potential study participants to ensure they have preexisting opioid dependence in withdrawal studies and is also recommended by FDA in human abuse potential studies involving recreational drug users to determine if physical dependence is present (FDA, Jasinski et al., 1984; 2017).

c Adverse event assessment including drug-specific scales for assessing withdrawal (e.g., Subjective Opiate Withdrawal Scale (SOWS) and Clinical Opiate Withdrawal Scale (COWS) are included in clinical safety and efficacy studies to determine if withdrawal emerges upon discontinuation of drug treatment (Huestis et al., 2022; FDA, 2017; Vicknasingam et al., 2020).

d There are drugs that do not produce cross dependence but suppress spontaneous withdrawal symptoms (e.g., clonidine and lofexidine suppress opioid withdrawal, and varenicline reduces nicotine withdrawal).

Because some people who develop kratom withdrawal do seek help in its management, it is important to evaluate potential non-opioid approaches to providing relief of kratom withdrawal including lofexidine, clonidine, and gradual kratom and mitragynine dose tapering (“weaning”). This might also include evaluation of behavioral interventions to help sustain remission from kratom dependence, in cases where it does occur, in lieu of long-term buprenorphine or methadone regimens. In-so-far as preliminary survey and anecdotal evidence suggests that kratom use disorder and physical dependence appears more likely in people with preexisting opioid use and efforts to self-manage opioid withdrawal and OUD, it is important to better understand the factors that influence kratom dependence in the absence of opioid use. It is necessary to better characterize individual differences – risk and protective factors – associated with development of kratom withdrawal upon cessation of use.

## 4 Conclusion

From a neuropharmacological perspective, kratom has emerged as a fascinating area of exploration already helping to better understand how substances providing pain relief at least partially modulated by morphine opioid receptor pathways can differ so widely in their effects related to abuse potential, physical dependence, withdrawal related effects, and respiratory effects (e.g., Henningfield et al., 2022; Qu et. al. 2022). Research is rapidly advancing our knowledge, from a public health and societal perspective, this research is urgent because the toll of opioid overdose deaths continues to increase and is not likely to dramatically reverse in the near term. Such research may have short term implications for kratom-related policy and regulation to prevent adulterated products, as well as longer term implications for the potential development of new generations of medicines with improved safety and efficacy for a variety of CNS-related disorders.

## Role of funding Source

JEH and DWW are employees of PinneyAssociates which partially supported their efforts on this article and paid the OpenAccess fee. KES reports that support was provided by the Intramural Research Program of the National Institutes of Health, NIDA, and affirms that the opinions expressed in this article are the authors’ own and do not reflect the view of the NIH, the Department of Health and Human Services, or the United States government. The following authors report nothing declared: MCC, OG, NH, ZH, LRM, AGR, AS, DS, MTS, and ZW.

## Contributors

JEH, MAH and DWW developed the initial draft of this article based on presentations and discussion in the Forum then circulated it to all coauthors for review and editing. This was an interactive process over several months involving several opportunities for review, revisions and editing by all authors.

## Declaration of Competing Interest

JEH, MAH and DWW provide scientific and regulatory advising on new medicines, dietary supplements, cannabinoids, and tobacco/nicotine products for FDA regulation, through PinneyAssociates, and MAH also provides forensic toxicology and related advising independently of PinneyAssociates. The following authors report no conflict declared: MCC, OG, NH, ZH, LRM, AS, DS, KES, MTS, and ZW. AGR reports serving as a paid scientific advisor to NeonMind Biosciences, InnerWell, and ETHA Natural Botanicals.
